# Transparency and Variability in Pricing for Pediatric Outpatient Imaging in US Children’s Hospitals

**DOI:** 10.1001/jamanetworkopen.2022.0736

**Published:** 2022-03-02

**Authors:** Shireen E. Hayatghaibi, Vinicius V. Alves, Rama S. Ayyala, Jonathan R. Dillman, Andrew T. Trout

**Affiliations:** 1 Department of Radiology, Cincinnati Children’s Hospital Medical Center, Cincinnati, Ohio; 2 Cincinnati Children's Hospital, Cincinnati, Ohio; 3 Department of Radiology, University of Cincinnati College of Medicine, Cincinnati, Ohio; 4 Department of Pediatrics, Cincinnati Children’s Hospital Medical Center, Cincinnati, Ohio

## Abstract

This cross-sectional study assesses price transparency compliance and price variability for outpatient imaging services among top US children’s hospitals.

## Introduction

Rising out-of-pocket health care spending and the expansion of high-deductible health plans have increased the cost burden for patients.^1^ In January 2021, the Hospital Price Transparency Rule was implemented, requiring hospitals to (1) publish chargemaster rates, discounted cash prices, and payer-negotiated prices in a machine-readable file; and (2) publish costs for 300 common shoppable medical services in a consumer-friendly format.

Medical service price disclosure is intended to tame price growth. This is particularly relevant to medical imaging, which is commonly delivered in an outpatient setting and is costly, creating an environment where patients may decide where to consume based on price. We assessed price transparency compliance and price variability for outpatient imaging services among top US children’s hospitals.

## Methods

This cross-sectional study was not human research and, as such, did not require institutional review board approval or informed patient consent, in accordance with 45 CFR §46. The study followed the Strengthening the Reporting of Observational Studies in Epidemiology (STROBE) reporting guideline.

This study included all children’s hospitals ranked by *US News and World Report* from 2021 to 2022 (n = 89). Data were collected from September to October 2021 through a manual search of hospital websites using keywords *price* and *transparency*. Compliance was assessed for both price transparency rule requirements. Hospitals were coded as fully compliant if they met both parameters.

Price variance analysis was performed for 10 outpatient imaging examinations, encompassing multiple imaging modalities and examinations with high-volume outpatient pediatric utilization. Among compliant hospitals, mean charges, cash prices, payer negotiated prices, number of payer plans, and the coefficient of variance (CoV) were calculated. The markup from Medicare maximum reimbursement was calculated based on mean negotiated rates.

## Results

Of the 89 sampled hospitals, 98% (n = 87) were compliant with the shoppable services requirement, but only 39% (n = 35) were fully compliant with both requirements. The most common deficiency was related to the machine-readable file element. Among sampled hospitals, 53% (n = 47) omitted minimum and maximum negotiated rates, 51% (n = 45) omitted payer negotiated rates, 40% (n = 36) omitted cash prices, and 9% (n = 8) omitted chargemaster rates.

Among fully compliant hospitals, the greatest cash price variation was for retroperitoneal ultrasound (CoV: 84%), computed tomography (CT) of the head without contrast (CoV: 82%), and complete abdominal ultrasonography (CoV: 74%). Charge variation for these examinations was less (retroperitoneal ultrasonography: 45%; head CT without contrast: 53%; and complete abdomen ultrasonography: 48%) (Figure). The greatest markup from mean negotiated prices compared with Medicare reimbursement was for magnetic resonance imaging (*Current Procedural Terminology [CPT]* code: 70551 [$1693 vs $230], 74183 [$2381 vs $368], 73721 [$1539 vs $230]) and CT (*CPT *code: 70450 [$1028 vs $109], 74177 [$2265 vs $368]) (Table).

**Figure.**
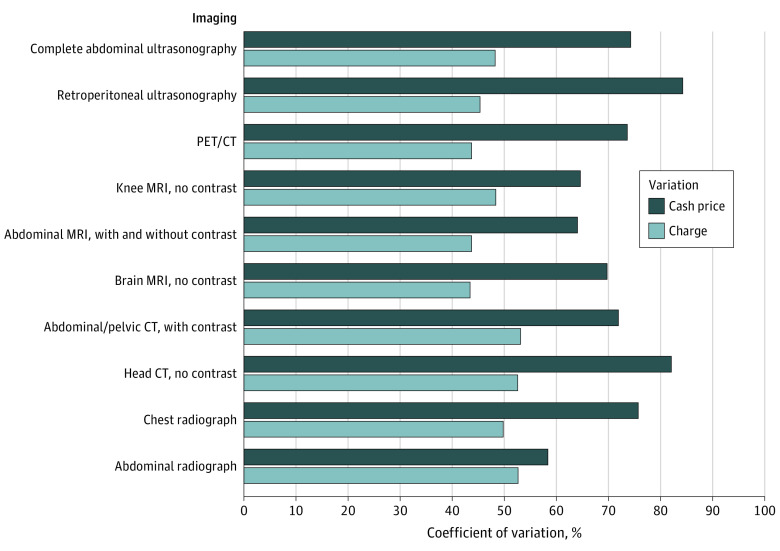
Charge and Cash Price Variation for 10 Common Outpatient Imaging Examinations CT indicates computed tomography; MRI, magnetic resonance imaging; PET, positron emission tomography.

**Table.** Prices of 10 Common Outpatient Imaging Examinations Across 35 Children’s Hospitals

Description	*CPT* code	$, mean (SD) [range]					Medicare rate, $
		Charge^a^	Cash price	Negotiated price		No. of plans	
				Minimum^b^	Maximum^c^		
Radiography							
Abdominal	74018	397 (209) [118-788]	209 (122) [0-506]	73 (62) [0-244]	306 (152) [76-670]	48 (83) [4-473]	81
Chest	71046	460 (229) [120-891]	251 (190) [0-833]	76 (70) [0-319]	367 (187) [83-802]	48 (83) [4-473]	81
CT							
Head without contrast	70450	2064 (1085) [515-5000]	1199 (984) [0-5000]	308 (338) [0-1349]	1748 (838) [369-3700]	48 (83) [4-473]	109
Abdomen and pelvis with contrast	74177	4658 (2478) [1608-13768]	2626 (1888) [0-7813]	629 (809) [0-4268]	3900 (2632) [106-11703]	48 (83) [4-473]	368
MRI							
Brain without contrast	70551	3467 (1505) [1270-7671]	2081 (1451) [0-7091]	496 (576) [0-2378]	2890 (1449) [333-6520]	48 (83) [4-473]	230
Abdomen with and without contrast	74183	5083 (2219) [1957-11870]	2988 (1914) [0-9474]	701 (825) [0-3680]	4060 (2370) [340-10809]	48 (83) [4-473]	368
Knee without contrast	73721	3315 (1604) [1260-9186]	1929 (1246) [0-5318]	458 (519) [0-2378]	2619 (1456) [161-6521]	48 (83) [4-473]	230
PET/CT	78816	8045 (3518) [3618-19575]	4790 (3513) [0-14459]	1329 (1142) [0-4814]	6407 (3059) [73-14771]	48 (83) [4-473]	1481
Ultrasonography							
Retroperitoneal	76770	1078 (489) [166-4241]	717 (604) [0-2995]	318 (352) [0-2032]	941 (620) [120-3605]	48 (83) [4-473]	109
Abdominal complete	76700	1219 (588) [181-4034]	719 (534) [0-2584]	185 (185) [0-654]	1076 (734) [147-3429]	48 (83) [4-473]	109

Abbreviations: *CPT, Current Procedural Terminology*; CT, computed tomography; MRI, magnetic resonance imaging; PET, positron emission tomography.

^a^ Mean charge includes 81 hospitals that published charges.

^b^ Minimum negotiated price is the mean of minimum payer negotiated rate taken for the included hospitals.

^c^ Maximum negotiated price is the mean of maximum payer negotiated rate taken for the included hospitals.

## Discussion

Our study contributes to reports on health care price transparency and documents low compliance and high variability of imaging examination prices at pediatric institutions. Only 39% of sampled hospitals were fully compliant with the Hospital Price Transparency Rule. Most hospitals complied with the shoppable services requirement, providing patients the ability to price shop for a fixed number of services. However, there are limited data suggesting that patients will access price information and select care based on price.^2^

A limitation of this study was the sample only included top pediatric hospitals, which may not be representative of all children's hospitals. Although our study is focused on top pediatric hospitals, our results are consistent with prior reports that found most hospitals deficient in the machine-readable file stipulation.^3,4,5,6^ Additionally, imaging prices did not include the professional component for examination interpretation, which further restricts the effectiveness of the transparency policy.

Despite the type of imaging examination, prices in pediatric hospitals vary widely. Variation is reflected in charges for imaging services, discounted cash prices, and in negotiated prices. By example, the mean maximum negotiated amount for a CT of the abdomen and pelvis was 6 times the mean minimum negotiated amount. We also found greater variation in cash prices compared with imaging charges, which can have substantial ramifications for uninsured patients.
